# Genetic Correlations between Boar Taint Compound Concentrations in Fat of Purebred Boars and Production and Ham Quality Traits in Crossbred Heavy Pigs

**DOI:** 10.3390/ani13152445

**Published:** 2023-07-28

**Authors:** Sara Faggion, Paolo Carnier, Valentina Bonfatti

**Affiliations:** Department of Comparative Biomedicine and Food Science, University of Padova, Viale dell’Università 16, 35020 Legnaro, Italy; sara.faggion@unipd.it (S.F.); paolo.carnier@unipd.it (P.C.)

**Keywords:** indole, skatole, androstenone, carcass, dry-cured ham, genetic correlation

## Abstract

**Simple Summary:**

Boar taint (BT) is an off-odor and off-flavor affecting pork caused by the accumulation in the tissues of uncastrated male pigs of three main compounds: androstenone, skatole, and indole. The reduction in BT compound accumulation by genetic selection is a viable strategy to stop surgical castration of male piglets. Understanding the genetic relationships between BT compound concentration and carcass and meat quality is necessary to assess the potential effects of selecting against BT on other traits of interest. In pigs, selection is operated in purebred lines, but slaughter pigs are mostly crossbred. Therefore, before selecting against BT, it is crucial to evaluate the potential effects of such selection on commercial traits measured in crossbreds. The moderate heritability values estimated for BT compound concentrations confirmed that breeding strategies against BT can be successful. Results indicated that there is a correlation between BT compound concentrations measured in purebred pigs and carcass and ham quality traits of crossbreds. In particular, selection against BT might lead to a favorable increase in carcass backfat depth. However, such selection is also expected to negatively impact dry-cured ham quality through decreased ham fat thickness and increased proportion of unsaturated fatty acids in ham subcutaneous fat.

**Abstract:**

Selecting pigs with reduced ability to accumulate boar taint (BT) compounds in their tissues is an alternative to male surgical castration. As the majority of slaughter pigs are crossbred, before selecting against BT in purebreds, it is essential to consider possible impacts on commercial traits in crossbreds. This study estimated the genetic correlations between BT compound levels measured in 1115 purebred pigs and carcass and ham quality traits collected in 26,577 crossbred Italian heavy pigs. Genetic correlations were estimated in bivariate Bayesian analyses including one BT trait and one production or ham quality trait at a time. Heritability of androstenone, skatole, and indole was 0.41, 0.49, and 0.37, respectively. A moderate negative correlation between skatole and carcass yield (−0.40), and between all BT compounds and backfat (from −0.26 to −0.55) was observed. Conversely, positive correlations (from 0.11 to 0.54) were found between skatole and ham fat thickness traits. Correlations between BT compounds and iodine number ranged from −0.07 (for androstenone) to −0.64 (for skatole), whereas those with PUFA ranged from −0.13 (for indole) to −0.33 (for skatole). Hence, reducing BT could decrease ham fat thickness and increase unsaturated fatty acids, with potential negative impacts on product quality.

## 1. Introduction

With the current and perspective future expansion of the ban on surgical castration in Europe, a gradual introduction of immunocastrated or entire male pigs is taking place [1,2], resulting in improved animal welfare, better feed conversion, and higher lean deposition [3]. However, immunocastrated pigs may raise concerns among consumers, whereas entire male pigs are characterized by a more aggressive behavior and the potential occurrence of boar taint (BT), an unpleasant odor and taste that can be detected in their meat [3,4]. Although other substances also contribute to its perception [5,6], BT is primarily caused by the accumulation in fat tissue of androstenone (5-androst-16-en-3-one, AND), a steroid hormone produced in the testes, as well as indole (2,3-benzopirrole, IND) and skatole (3-methylindole, SKA), two fecal compounds derived from tryptophan degradation operated by gut bacteria. The accumulation of these compounds (hereafter referred to as “BT compounds”) increases with the surge in plasma steroid hormones; thus, the risk of BT grows when approaching sexual maturity [7,8,9].

Genetic selection to decrease BT is considered a safe and cost-effective strategy to stop surgical castration, with long-term cumulative effects [3]. Selection against BT is expected to exert limited effects on growth and finishing performance [10,11], as suggested by the low or trivial genetic correlation between BT compound concentrations and those traits. However, when compared to surgically castrated pigs, entire males exhibit altered meat quality (i.e., lower adiposity, differences in water holding capacity, and higher meat toughness). Hence, the use of entire pigs with a genetically low incidence of BT might affect meat processing and final product quality [12].

In Italy, the pig breeding industry is largely oriented to the production of Protected Designation of Origin (PDO) dry-cured ham [13]. On the whole, Italy produces about 12 million PDO dry-cured hams per year [14] that should be added to many other cured products processed from different cuts, with a total economic value of more than 3 billion EUR per year [15]. To ensure high-quality curing, pigs are slaughtered at an age of not less than 9 months and a carcass weight of 110–168 kg [16], and they are subject to specific selection plans that aim at enhancing fat deposition. Under these circumstances, a negative genetic correlation between BT compound concentrations and carcass leanness traits, consistently reported in the literature [3,17], would be unfavorable. Understanding the genetic relationships between BT compound concentration and carcass and meat quality is needed to evaluate the possible effects of selecting against BT on other economically important traits.

To take advantage of both additive and nonadditive genetic effects, crossbreeding is widely adopted in pigs [18], and nearly 80% of hams processed into PDO dry-cured hams are from three- or four-way crossbred (CB) animals [19]. In this scenario, where the economic benefits of pig production rely on CB performance, the effects on CB traits due to selective breeding implemented in purebred (PB) lines to reduce BT deserve attention. Even though exploitable additive genetic variation in BT exists for selection purposes, the success of a breeding program depends on the genetic relationships between PB BT traits and CB performance.

Existing estimates of the effects of BT selection strategies on production and meat quality traits are contradictory, and all the existing reports have focused on entire PB or CB pigs slaughtered at 100–110 kg BW [17,20], and no studies have been conducted on heavy pigs. In addition, the genetic relationship between BT compound concentration and ham quality traits is currently unknown. Hence, the aim of the study was to investigate the genetic relationships between concentrations of AND, SKA, and IND in adipose tissue of PB boars and carcass and ham quality traits measured in CB heavy pigs.

## 2. Materials and Methods

Data were collected from two locations: a nucleus farm, where purebred C21 boars are produced and boars selected as nucleus sires are mated to purebred C21 nucleus sows, and a sib testing station, where semen from C21 nucleus boars is used to inseminate crossbred sows to produce approximately 35 crossbred piglets per each nucleus boar. The hybrid dams originated from a cross involving boars of a synthetic line, derived from Large White and Pietrain breeds, and sows of a Large White line selected for maternal ability and prolificacy. Selection of C21 breeding candidates is based on estimates of genetic merit obtained from (i) their own phenotypes for growth performance, and (ii) phenotypic data on carcass and ham quality provided by their CB half-sibs raised under commercial conditions in the sib testing farm. 

### 2.1. Purebred Animals

A fat biopsy was collected from 1115 young boars of the PB C21 Goland sire line (Gorzagri, Fonzaso, Italy) from January 2020 to June 2022. Pigs were offspring of 27 nucleus boars and 226 nucleus sows. Pigs were reared at the same farm and fed ad libitum using 14.4% CP and 3.07 kcal ME/kg.

Biopsy collection and quantification of AND, SKA, and IND were performed as described by Boschi et al. [21]. Briefly, animals were subjected to local anesthesia, and a backfat biopsy sample (~0.5 g) was collected in vivo from the neck area of each pig at 209 ± 13 days of age (153.4 ± 15.5 kg of BW) using a biopsy device (SUISAG, Sempach, Switzerland). Fat samples were stored at −80 °C until laboratory analyses. The concentration of AND, IND, and SKA was assessed by reversed-phase HPLC with fluorescence detection. Two different chromatographic separations, one for IND and SKA, and one for AND, were performed. Androstenone concentration was quantified after sample derivatization. Details on sample preparation, chromatographic conditions, and accuracy of the method can be found in Boschi et al. [21].

### 2.2. Crossbred Animals

Individual phenotypes on growth performance, as well as carcass and ham quality traits, were from 26,577 CB finishing pigs produced in the sib-testing program of the C21 Goland sire line (Gorzagri, Fonzaso, Italy). Pigs (13,139 barrows and 13,438 gilts) were the progeny of 731 C21 Goland boars mated to 1885 CB sows. Crossbred piglets were born between February 1998 and July 2021. In the growth and finishing phase, pigs were managed under consistent conditions, as described by Rostellato et al. [11], and slaughtered at 274 ± 9 days of age and 167.9 ± 24.3 kg BW, at the same abattoir, in groups of about 70 animals each.

Final BW was adjusted to 270 days using individual linear regressions of BW on age estimated from 6 BW measures (at 60, 90, 135, 180, and 245 days of age and the day before slaughter). Hot carcass weight was recorded online, while measures of carcass backfat and loin depth were estimated by image analysis of the left carcass side (CSB-Image-Meater^®^, CSB-System AG, Geilenkirchen, Germany) and used to estimate carcass lean meat content according to EU guidelines [22,23].

The following phenotypes of green ham (i.e., the raw ham before dry-curing) were also recorded, as detailed in Bonfatti et al. [24]: visible/near-infrared predicted fat quality including iodine number and fatty acid composition of subcutaneous fat (C18:2n6, C18:0, PUFA, and MUFA-to-PUFA ratio, determined as in Bonfatti et al. [25]); ham subcutaneous fat depth measured in the proximity of *biceps femoris* and *semimembranosus* muscles; ham fat cover thickness (linear score, from −4 = very thin to 4 = very thick); round shape (linear score, from 0 = low roundness to 4 = high roundness); marbling of the visible muscles of the thigh (linear score, from 0 = absent to 4 = evident marbling); visible muscle color (linear score, from −4 = very pale to 4 = very dark); veining (linear score, from 0 = absent to 4 = very evident); visible/near-infrared predicted ham weight loss (WL, %) after 12 months of dry-curing, obtained by the method described by Bonfatti and Carnier [26].

### 2.3. Statistical Analysis

To normalize the distribution of AND, SKA, and IND content, variables were log-transformed before the statistical analysis. Additive genetic relationships among animals were computed on the basis of a minimum of six generations of known ancestors. Sires and dams of CB pigs were unrelated. The pedigree included 33,629 records from 1144 sires and 4585 dams.

(Co)variance components for the investigated traits were estimated in a set of Bayesian bivariate analyses using the Markov chain Monte Carlo (MCMC) method of Gibbs sampling implemented in GIBBSF90 of the BLUPF90 family of programs [27]. Each bivariate analysis included a PB BT trait and a CB production or ham quality trait at a time. In addition to the random additive genetic effect of the animals, models for BT traits included the systematic effect of the date of analysis (94 levels), whereas, for production and ham quality traits, the model included the effects of sex (female and castrated male) and slaughter group (from 106 to 367 levels, depending on the trait). As no PB pig had a record for carcass and meat quality traits, the residual covariances between PB traits and CB traits were constrained to 0.

Flat priors were assumed for the systematic effects and the covariance components [28]. The prior distributions of the additive genetic effects and residuals were multivariate normal distributions. The conditional posterior distributions of covariance components of the additive genetic and residual (co)variance matrix were inverse Wishart distributions.

The marginal posterior distribution of the genetic and environmental effects was estimated from a Gibbs chain of 1,000,000 samples of the parameters, where the initial 100,000 samples were discarded (burn-in), and a lag of 20 iterations was applied to reduce the autocorrelations between consecutive iterations. The convergence of the Markov Chain was tested using the algorithms of Raftery and Lewis [29].

### 2.4. Estimates of (Co)Variance Components and Genetic Parameters

Numerical integration through the Gibbs sampler was used to estimate the marginal posterior densities of (co)variance components and genetic parameters. The posterior median was used as a point estimate of (co)variance components and parameters thereof. The lower and upper bounds of the highest 95% posterior probability density interval (HPD 95%) for (co)variance components and genetic parameters were obtained from the estimated marginal densities using POST-GIBBSF90.

The probability of the estimated genetic correlation being >0 for positive estimates or, alternatively, being <0 for negative estimates (P_0_) was calculated using the R software [30]. Similarly, the probability of the estimated genetic correlation being >0.1 for positive estimates or, alternatively, being <−0.1 for negative estimates (P_01_) was also calculated. Correlations were considered to be different from zero if P_0_ was >0.80, and they were considered to be relevant if P_01_ was >0.70.

Heritability of a trait was defined as

(1) $$h^{2}=\sigma_{a}^{2}/(\sigma_{a}^{2}+\sigma_{e}^{2}),$$

where $\sigma_{a}^{2}$ is the additive genetic variance, and $\sigma_{e}^{2}$ is the residual variance.

## 3. Results and Discussion

### 3.1. Descriptive Statistics for the Investigated Traits

Summary statistics for the BT compound concentrations and CB traits are reported in Table 1. Means of AND, SKA, and IND were in the lower range reported in the literature [31,32,33,34]. The number of observations for carcass and ham quality traits was variable across traits because collection of individual phenotypes started at different times for different traits. The number of individual records ranged from 7653 for carcass lean meat content to 26,238 for ham round shape score. Means of BW at 270 days, iodine number (<70), linoleic acid (<15%), and subcutaneous fat depth (>20 mm) for the sample investigated in this study were consistent with the requirements of PDO product specifications [35,36].

### 3.2. Genetic Parameters of Boar Taint Compound Concentrations

Boar taint compound concentrations were moderately heritable (Table 1): heritability estimates for BT traits ranged from 0.37 (for IND) to 0.49 (for SKA) and were within the range reported in the literature. In recent studies, most estimates of the genetic parameters were obtained for Landrace, Large White, Duroc, and Pietrain breeds and ranged from 0.4 to 0.6 for AND, and from 0.3 to 0.6 for SKA [3]. Estimates for IND are scarce, but generally comparable with or higher than the range obtained in this study [10,31].

The estimated genetic correlations (r_g_) among BT compound concentrations were all positive and ranged from moderate to high (Table 2). In particular, the genetic correlation between AND and SKA was 0.40 (HPD 95%: 0.06–0.66). In agreement with our results, in the literature, the genetic correlation between backfat AND and SKA contents is reported to be positive, with estimates ranging from 0.2 to 0.6 [3]. Consistent with findings of Windig et al. [10] and Baes et al. [31], the estimated genetic correlation between SKA and IND was positive and high (r_g_ = 0.85; HPD 95%: 0.69–0.99), indicating that selection strategies aiming at decreasing BT occurrence might efficiently reduce all BT compounds simultaneously. This can be ascribed to their common pathway; AND inhibits the synthesis of degradation enzymes for SKA and IND in the liver [37]. Furthermore, SKA, IND, and AND are partially degraded in both the liver and the kidney [38], and SKA and IND follow the same pathway for both synthesis and degradation [39].

### 3.3. Genetic Correlation between Boar Taint Compounds and Growth Rate or Carcass Leanness

Genetic correlations between BT compound concentrations and carcass and ham quality traits are reported in Table 2. The majority of the estimated genetic correlations were weak and exhibited wide HPD 95% intervals, which can be ascribed to the small sample size for BT compound concentrations and to the limited connection between the PB and CB populations (only 27 sires had both PB with BT records and CB offspring, in the years between 2020 and 2022). Genetic correlations between BT compound concentrations and BW at 270 days (−0.11, −0.07, and −0.19 for AND, SKA, and IND, respectively) were not statistically different from zero. Hence, a decrease in BT in PB is not expected to affect CB growth rate. In line with our results, genetic correlations between AND or SKA and growth rate, measured as either average daily gain or BW at fixed age, were reported to range from −0.23 to 0.04 [10,20,33,40,41]. The genetic correlation with carcass yield was not consistent across BT compounds, being not different from zero for AND and IND, but negative for SKA (−0.40, P_01_ = 0.92), indicating that a reduction in SKA is expected to favorably affect this trait.

Boar taint compound concentrations exhibited a negative genetic relationship with carcass backfat. Genetic correlations were −0.26, −0.55, and −0.44, for AND, SKA, and IND, respectively, with estimates of P_01_ equal to 0.80, 0.93, and 0.95, respectively. Consistently, the correlation between AND and ham marbling score was equal to −0.26 (P_01_ = 0.89). On the other hand, the genetic correlation between BT and carcass lean meat content was not different from zero. This indicates a negative genetic relationship between BT and fat accretion, but no association between BT and lean tissue accretion. In a genome-wide association study performed on the same pig line used in this study, genes related to adipogenesis have been reported to be associated to BT compound concentrations [42].

Although the estimated correlations indicate that a reduction in BT compound concentration in PB by genetic selection would result in an increase in carcass backfat thickness in CB, an opposite effect is expected on ham subcutaneous fat thickness. Indeed, BT compounds exhibited a positive (SKA) or a negative/null (AND and IND) correlation with ham fat thickness score and fat thickness measured near the *biceps femoris* muscle. In addition, AND and SKA were also positively correlated to ham fat depth at the *semimembranosus* muscle (r_g_ = 0.16 and 0.43, respectively; P_01_ = 0.66 and 0.99, respectively). Hence, with a decrease in BT due to genetic selection, ham fat thickness is expected to decrease near both the *biceps femoris* and the *semimembranosus* muscle.

According to the estimated genetic correlations between production and ham quality traits (data not reported in tables), carcass backfat thickness exhibited a stronger correlation with the subcutaneous fat thickness near the *biceps femoris* muscle (r_g_ = 0.57) than near the *semimembranosus* muscle (r_g_ = 0.20). Additionally, the genetic relationship between the depth of fat measured at the two muscles was low (r_g_ = 0.29). In a previous study conducted on the same pig population [43], the genetic correlation between the average ham subcutaneous fat thickness and the extent of the “fat-eye” depot (i.e., an intermuscular fatty area delimited by *gastrocnemius*, *biceps femoris*, *semitendinosus*, and *semimembranosus* muscles) was also low (r_g_ = 0.28).

These results seem to indicate that decreasing levels of BT compound concentrations are associated with a different distribution of the subcutaneous fat across anatomic regions. To the best of our knowledge, no studies have been conducted on the genetic relationships between ham fat deposition and BT compounds, but we might argue that lipogenesis/lipolysis occurs at different rates across body regions.

The subcutaneous fat at the *semimembranosus* muscle is significantly thinner than that near the *biceps femoris*. In particular, the anatomic region in proximity of the *semimembranosus* muscle, at which fat thickness was measured, is the region where fat depth is at its lowest. As ham subcutaneous fat thickness modulates salt penetration and water losses during dry-curing [13], it plays a critical role in determining the seasoning aptitude of hams [26]. In the investigated pig population, the genetic correlation between fat depth measured at *biceps femoris* and *semimembranosus* muscle and ham weight loss during dry-curing was −0.74 and −0.43, respectively (results not reported in tables). Even though a reduced fat thickness nearby the *biceps femoris* muscle is more desirable by consumers and may improve ham’s marketability [44], a decrease in fat depth at this site might impair ham technological properties, with possible unfavorable effects on product quality.

The BT compound exhibiting the largest correlations with both carcass and ham fat thickness was SKA, which is the compound that most influences BT perception [17]. Hence, a breeding program targeting SKA to reduce the risk of BT occurrence would need to overcome the challenge of the unfavorable correlation between the concentration of this compound and ham fat coverage.

Contrarily to our results, literature estimates of the genetic correlations between carcass backfat thickness and BT are consistently positive, indicating that selection to reduce BT would result in leaner carcasses. A positive relationship between AND and carcass fatness was also observed in a divergent selection experiment [45], where greater AND concentrations were detected in boars selected for increased fatness and low growth rate compared with boars selected for increased leanness and high growth rate. Likewise, the correlation of carcass leanness, measured by loin muscle area, or carcass meat percentage, was consistently reported to be moderate and negative for AND (r_g_ = −0.26 to −0.10) and SKA (r_g_ = −0.24 to −0.10) [3]. When different breeding scenarios were compared, Haberland et al. [46] observed a decrease in BT even when BT was not in the breeding goal, due to a favorable correlation between BT reduction and the desired change for other traits, namely, lean meat percentage and feed conversion rate.

However, the large majority of previous studies estimated genetic correlations in entire male pigs of 100–110 kg BW belonging to lines selected for lean growth, with records on both BT compound concentrations and production traits. Furthermore, when BT and production traits were not recorded on the same animal, different animals of the same genetic background and sex were used to estimate the genetic correlations between traits. The current breeding goals of the pig line used in this study focus on the enhancement of growth performance, as well as ham and carcass quality traits. In particular, selective breeding aims at enhancing the quality of the green ham and its suitability for dry-curing, which largely depends on fat deposition. These breeding objectives, different from those of populations involved in previous studies, impact adipogenesis, which is known to be related, among others, to the hormonal status of the animal [47].

In addition, in our study, genetic correlations were estimated between animals of different genetic background (PB vs. CB) and different sex (entire males vs. females and barrows), raised in different conditions (nucleus vs. commercial farm), and fed using different feeding strategies (*ad libitum* vs. restricted). The CB pigs used in our study had a lower ratio of lean-to-fat gain compared to pigs used in previous studies because they were slaughtered when their body accretion in the finishing phase was largely represented by fat deposition. As a biological relationship exists between steroid hormones and body fat deposition [48], the body composition of CB might influence the association between CB performance traits and PB BT compound concentration.

While, in previous literature studies, the genetic correlations between BT traits and performance and production traits were estimated in entire males, the CB data used in this study were collected from females and barrows. It is known that some traits have a sex-dependent expression [49]. If sex differences in phenotype result from genotype by sex interaction, genetic correlation between sexes significantly differs from unity. For example, Saintilan et al. [50] estimated genetic correlations equal to 0.65 and 0.69, respectively, between castrates and entire males or females for backfat thickness. A genotype by sex interaction cannot be excluded for the traits investigated in the present study and might partially explain the inconsistencies between our estimates and those obtained by other authors. However, this hypothesis cannot be proven as no entire male pigs with carcass and ham quality records are available.

### 3.4. Genetic Correlation between Boar Taint Compound Levels and Fatty Acid Profile 

The estimated genetic correlations between BT compound levels and traits related to the concentration of PUFA in fat (iodine number, linoleic acid, and total PUFA content) were low and negative (Table 2). In particular, the strongest correlation was observed with SKA. In addition to the increased carcass backfat thickness and decreased ham fat thickness, selection to decrease BT in PB is also expected to increase the proportion of PUFA in fat of CB. The correlation of BT compounds with iodine number was −0.07 (P_0_ = 0.70), −0.64 (P_0_ = 1), and −0.54 (P_0_ = 1) for AND, SKA and IND, respectively. The genetic correlations with PUFA were −0.20 (P_01_ = 0.77) and −0.33 (P_01_ = 0.76) for AND and SKA, respectively, very similar to the estimates obtained for the correlations of the compounds with linoleic acid concentration. These estimates are consistent with the weak positive association (r_g_ = 0.02–0.25) detected between the compounds and stearic acid content, and the moderately high relationship between the compounds and the MUFA-to-PUFA ratio (r_g_ = 0.25–0.50).

Fatty acid composition and iodine number are particularly important for ham production because they affect the overall quality of the final product, as well as fat firmness and texture [13]. Although a high proportion of PUFA may be beneficial for human health, low concentrations of PUFA in fat of dry-cured products are desired to maintain high quality standards [51]. An increased proportion of PUFA in adipose tissue can result in poor fat firmness and tissue cohesion, as well as an increased risk of fat oxidation and peroxidation, which can cause rancidity and off-flavors. This is particularly problematic for products that require long curing periods, such as PDO dry-cured hams, which have specific thresholds in place for iodine number and linoleic acid [35,36].

The relationship between BT and fatty acid composition can potentially influence meat quality but, to date, specific studies on such relationship are scarce. Mörlein and Tholen [52] evaluated fatty acid composition in entire male pigs with highly divergent levels of BT compounds and observed that saturated fatty acids increased in males with high levels of AND and SKA, while PUFA increased in boars with low levels of AND and SKA, in agreement with our results.

### 3.5. Genetic Correlation between Boar Taint Traits and Other Green Ham Quality Traits

The estimated genetic correlations between BT compound concentrations and ham roundness were null for AND (r_g_ = 0.01) and SKA (r_g_ = 0.05), and positive for IND (r_g_ = 0.24, P_01_ = 0.75) (Table 2). Ham roundness is associated with muscularity, and it is scarcely studied in the literature. In general, a marked roundness is frequently associated with excessive leanness, insufficient fat covering, high ham weight loss, and excessive salt absorption during dry-curing, resulting in poor product quality [53]. Hence, a decrease in roundness is considered favorable, but it is expected to exert a limited effect on ham quality if counterbalanced by a thinner subcutaneous fat covering, especially in proximity of the *semimembranosus* muscle.

Weak correlations between BT compounds and color were observed. Such correlations suggest that darker muscle color in CB hams is associated with high levels of AND in PB (r_g_ = 0.16, P_0_ = 0.85). The association between the levels of indolic compounds and color were slightly negative, but not statistically different from zero. Color score might be related to muscle pH and water holding capacity. As the desired color score is 0 (not too pale nor too dark), very close to the current average (−0.04), these correlations can be considered mildly unfavorable.

Veining is a visual defect of hams consisting in a visible subcutaneous venous lattice affecting the medial, or sometimes the entire, surface of the thigh. The estimated genetic correlations of BT traits with veining were not different from zero for AND and SKA, but a moderate positive correlation was observed between veining and IND (r_g_ = 0.25, P_01_ = 0.83). Hence, a decrease in BT would be beneficial for the reduction in veining defect. In CB pigs, veining was weakly but negatively associated with fatness traits (data not reported in tables), and this might explain the association with IND.

Despite the unfavorable correlation between BT compound concentrations and ham fat thickness, the genetic correlation of BT compounds with ham weight loss was not different from 0 (r_g_ ranged from 0.01 to −0.13; P_0_ from 0.52 to 0.71). This indicates that selection against BT is not expected to have negative effects on ham weight loss during curing.

## 4. Conclusions

The estimated heritability values for BT compound concentrations were moderate, confirming the opportunity of developing breeding programs to decrease BT. Estimating the genetic parameters for a large number of carcass and ham quality traits provided new insights into correlated effects affecting crossbred pigs and resulting from selection against BT operated in purebreds. Despite wide intervals obtained for some of the estimated correlations, our results indicate that a relationship between BT and carcass and ham quality traits exists. In particular, selection against BT could result in a favorable increase in carcass fat deposition. On the other hand, however, ham fat thickness is expected to be reduced, and the proportion of polyunsaturated fatty acids is expected to increase, with possible unfavorable repercussions on product quality.

## Figures and Tables

**Table 1:** Descriptive statistics and heritability of the investigated traits.

	N	Summary Statistics				Heritability ^1^	
		Mean	SD	Min	Max	Median	HPD 95%
**Purebred**							
Androstenone, log(ng/g)	1091	6.76	0.76	4.39	9.10	0.41	0.28; 0.55
Skatole, log(ng/g)	1115	3.28	1.07	−1.05	6.79	0.49	0.33; 0.67
Indole, log(ng/g)	1115	2.62	0.92	0.24	5.25	0.37	0.23; 0.54
**Crossbred**							
Body weight at 270 days, kg	22,097	170.22	15.71	99.70	236.40	0.50	0.46; 0.54
Carcass traits							
Killing out percentage, %	21,657	82.85	1.62	78.00	87.00	0.29	0.26; 0.32
Backfat depth, mm	17,651	27.31	5.38	10.00	54.00	0.41	0.37; 0.45
Lean meat content, %	7653	50.65	3.67	38.59	61.82	0.45	0.39; 0.51
Ham fatness traits							
Marbling, score	26,236	1.40	0.86	0.00	4.00	0.42	0.39; 0.46
Subcutaneous fat depth, score	26,099	0.05	1.56	−4.00	4.00	0.39	0.36; 0.43
Fat depth at *biceps femoris* muscle, mm	23,372	22.61	7.55	8.00	52.00	0.39	0.35; 0.42
Fat depth at *semimembranosus* muscle, cm	17,185	0.59	0.10	0.24	1.09	0.35	0.31; 0.38
Ham subcutaneous fat composition							
Iodine number	23,914	69.01	3.68	51.40	79.93	0.43	0.39; 0.47
PUFA, %	18,477	15.08	2.10	7.59	23.90	0.44	0.40; 0.48
C18:2n6, %	18,477	13.00	1.87	6.43	20.73	0.44	0.40; 0.48
C18:0, %	18,477	11.59	1.69	5.86	18.72	0.43	0.40; 0.47
MUFA/PUFA	18,477	3.31	0.49	1.34	5.28	0.41	0.37; 0.45
Other ham quality traits							
Round shape, score	26,238	1.72	0.87	0.00	4.00	0.37	0.34; 0.40
Color, score	26,100	−0.04	1.37	−4.00	4.00	0.32	0.29; 0.35
Veining, score	26,237	1.15	0.88	0.00	4.00	0.23	0.21; 0.26
Weight loss during dry-curing, %	14,552	26.70	1.85	20.13	32.94	0.36	0.32; 0.41

^1^ Median: median of the posterior probability density; HPD 95%: lower and upper bound of the highest 95% posterior probability density interval.

**Table 2:** Estimated additive genetic correlations (r_g_) between boar taint compound concentrations in purebred pigs and production and ham quality traits in crossbred pigs ^1^.

Trait	Androstenone				Skatole				Indole			
	r_g_	HPD 95%	P_0_	P_01_	r_g_	HPD 95%	P_0_	P_01_	r_g_	HPD 95%	P_0_	P_01_
**Purebred**												
Androstenone					0.40	0.06; 0.66	0.99	0.95	0.57	0.28; 0.85	1.00	0.99
Skatole									0.85	0.69; 0.99	1.00	1.00
**Crossbred**												
Body weight at 270 days	−0.11	−0.43; 0.18	0.72	0.51	−0.07	−0.49; 0.54	0.60	0.44	−0.19	−0.49; 0.66	0.78	0.65
Carcass traits												
Killing out percentage	0.00	−0.39; 0.50	0.50	0.38	−0.40	−0.65; 0.05	0.95	0.92	0.21	−0.51; 0.75	0.72	0.62
Backfat depth	−0.26	−0.53; 0.26	0.85	0.80	−0.55	−0.84; −0.04	0.96	0.93	−0.44	−0.75; −0.10	0.99	0.95
Lean meat content	0.06	−0.26; 0.53	0.63	0.41	−0.20	−0.59; 0.35	0.71	0.65	−0.35	−0.63; 0.30	0.73	0.68
Ham fatness traits												
Marbling	−0.26	−0.50; 0.00	0.98	0.89	0.11	−0.32; 0.34	0.71	0.53	0.04	−0.43; 0.30	0.56	0.37
Subcutaneous fat depth	−0.14	−0.48; 0.33	0.63	0.54	0.11	−0.12; 0.35	0.82	0.52	−0.17	−0.42; 0.12	0.88	0.70
Fat depth at *biceps femoris* muscle	−0.15	−0.43; 0.17	0.81	0.64	0.54	0.08; 0.77	0.99	0.96	−0.07	−0.35; 0.29	0.68	0.43
Fat depth at *semimembranosus* muscle	0.16	−0.15; 0.47	0.81	0.66	0.43	0.20; 0.70	1.00	0.99	0.05	−0.53; 0.34	0.56	0.40
Ham subcutaneous fat composition												
Iodine number	−0.07	−0.38; 0.24	0.70	0.44	−0.64	−0.89; −0.37	1.00	1.00	−0.54	−0.83; −0.17	1.00	1.00
PUFA	−0.20	−0.47; 0.14	0.90	0.77	−0.33	−0.59; 0.33	0.84	0.76	−0.13	−0.43; 0.39	0.68	0.55
C18:2n6	−0.21	−0.49; 0.11	0.91	0.80	−0.32	−0.60; 0.31	0.84	0.76	−0.12	−0.44; 0.38	0.67	0.54
C18:0	0.02	−0.35; 0.39	0.53	0.36	0.25	−0.24; 0.66	0.69	0.62	0.21	−0.07; 0.77	0.89	0.68
MUFA/PUFA	0.41	−0.24; 0.62	0.82	0.79	0.50	0.09; 0.88	0.99	0.95	0.25	−0.02; 0.87	0.93	0.77
Other ham quality traits												
Round shape	0.01	−0.38; 0.52	0.52	0.35	0.05	−0.20; 0.45	0.61	0.41	0.24	−0.08; 0.60	0.87	0.75
Color	0.16	−0.10; 0.58	0.85	0.59	−0.13	−0.46; 0.28	0.73	0.58	−0.16	−0.57; 0.23	0.78	0.57
Veining	−0.08	−0.52; 0.28	0.64	0.48	−0.01	−0.49; 0.33	0.52	0.32	0.25	−0.08; 0.52	0.92	0.83
Weight loss during dry-curing	0.01	−0.33; 0.38	0.52	0.29	−0.13	−0.38; 0.33	0.71	0.56	−0.02	−0.38; 0.43	0.55	0.34

^1^ HPD 95%: lower and upper bound of the highest 95% posterior probability density interval for the genetic correlation; P_0_: probability of the correlation being >0 (for positive estimates) or <0 (for negative estimates). P_01_: probability of the correlation being >0.1 (for positive estimates) or <−0.1 (for negative estimates).

## Data Availability

Restrictions apply to the availability of these data. Data were obtained from Gorzagri (Fonzaso, Italy) and are available from the authors with the permission of Gorzagri.
